# Supplementary material for: Integrated analysis of the aqueous humor microbiome and lens capsule transcriptome in high myopia cataract: a pilot study
Source: Front Med (Lausanne). 2026 Jun 16;13:1845205. doi: 10.3389/fmed.2026.1845205 (PMC13314463; doi:10.3389/fmed.2026.1845205)
Supplement: Supplementary file 3 [file Data_Sheet_1.zip › 3.Beta_Diversity/Distance/C372089/binary_jaccard_distance.pdf]

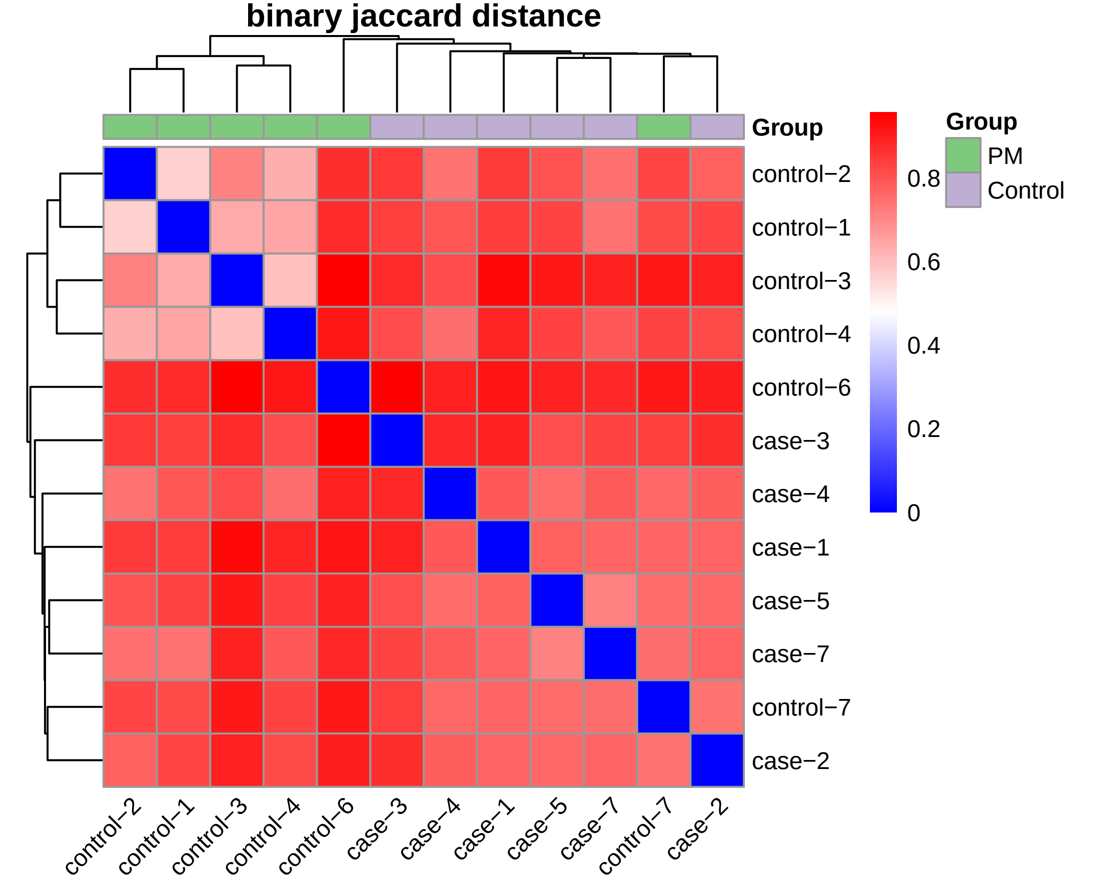

binary jaccard distance
Group
control−2
control−1
control−3
control−4
control−6
case−3
case−4
case−1
case−5
case−7
control−7
case−2
0.8
0.6
0.4
0.2
0
Group
PM
Control
